# GC–MS based untargeted metabolomics reveals the metabolic response of earthworm (*Eudrilus* *eugeniae*) after chronic combinatorial exposure to three different pesticides

**DOI:** 10.1038/s41598-023-35225-1

**Published:** 2023-05-26

**Authors:** Muneer Ahmad Malla, Anamika Dubey, Rajeesh Kumar Kori, Vandana Sharma, Ashwani Kumar, Shweta Yadav, Sheena Kumari

**Affiliations:** 1 Department of Zoology, Dr. Harisingh Gour University (A Central University), Sagar, MP 470003 India; 2 Metagenomics and Secretomics Research Laboratory, Department of Botany, Dr. Harisingh Gour University (A Central University), Sagar, MP 470003 India; 3 IRMS, National Dope Testing Laboratory, Ministry of Youth and Sports, GOI, New Delhi, India; 4 Quality Control & Quality Assurance Division, CSIR-Indian Institute of Integrative Medicine, Canal Road, Jammu, 180 001 India; 5 Metagenomics and Secretomics Research Laboratory, Department of Botany, University of Allahabad (A Central University), Prayagraj, UP 211002 India; 6 Institute for Water and Wastewater Technology, Durban University of Technology, Durban, 4001 South Africa

**Keywords:** Biological techniques, Biotechnology, Microbiology, Environmental sciences

## Abstract

In this study GC–MS-based untargeted metabolomics was used to identify the metabolic response of earthworm; *Eudrilus* *eugeniae* exposed to sub-lethal concentrations of chlorpyrifos-CHL, cypermethrin-CYP, Glyphosate-GLY, and Combined-C (all three pesticides) at the concentrations of 3, 6, and 12 mg/kg. Principal component analysis of the obtained datasets revealed a clear distinction between the control and treatment groups. The mean weight of the worms in the treated groups decreased significantly (*p* < 0.05). Among the identified metabolites, oleic acid (~ 93.47%), lysine (~ 92.20%), glutamic acid (~ 91.81%), leucine (~ 90.20%), asparagine (~ 94.20%), methionine (~ 92.27%), malic acid (~ 93.37%), turanose (~ 95.04%), maltose (~ 92.36%), cholesta-3,5-diene (~ 86.11%), galactose (~ 93.20%), cholesterol (~ 91.56%), tocopherol (~ 85.09%), decreased significantly (*p* < 0.05), whereas myoinositol (~ 83%) and isoleucine (78.09%) increased significantly (*p* < 0.05) upon exposure to the CHL, CYP, GLY, and C. Overall, the findings suggest that earthworms might be a new entry point for the pesticides into the food chain. The present study highlights that metabolomics can be a reliable approach to understand the effect of different xenobiotics including pesticides on the metabolic response of earthworms.

## Introduction

Organophosphate and pyrethroid pesticides are extensively used in agriculture to deal with different pests and safeguard crops^1,11^. However, overuse of these pesticides has emerged as a serious problem globally^2,–5^. Chlorpyrifos, cypermethrin, and glyphosate pesticides are persistent environmental contaminants of global concern due to an associated negative impact on agriculture and the environment^6,7^. These pesticides impair the central nervous system and cause neurodegenerative disorders in animals, including humans^8–10^. Therefore, chlorpyrifos, cypermethrin, and glyphosate are potential soil contaminants, and we must look at the effects of these pesticides on the soil environment^11–13^. Understanding and analyzing the environmental impacts of pesticides and their degradative metabolites in terrestrial ecosystems has become a significant precedence for Organization for Economic Cooperation and Development (OECD) and its fellow countries^14^.

Often representing the largest (> 80%) of soil biomass^15^, earthworms act as ecosystem engineers and play an essential role in key ecosystem processes^16^. They represent a prime example of a keystone species^17^ and experience persistent pesticide interactions in the soil environment, posing a serious threat to their lives^18^. Therefore, understanding the biological responses of earthworms to different environmental contaminants, including pesticides, is paramount importance for assessing soil and environmental health^19–21^. In agricultural soils, pesticides rarely occur as single individual compounds, instead, they exist as mixtures with different combinations^20–23^. To date, most studies on the toxicological effects have mainly focused on single compounds^24–27^. For example, Ratnasekhar et al. investigated the effect of cypermethrin on earthworms (*Metaphire* *posthuma*)^24^, and found that carbohydrates and lipids were mainly perturbed on exposure to cypermethrin. Similarly, Wang et al. evaluated the metabolic response of earthworm (*Perionyx* *excavatus*) to triphenyl phosphate^26^, and the authors reported significant perturbations in glucose, amino acids, inosine and phospholipids. In another study, Griffith et al. investigated the metabolic impacts of chlorothalonil in earthworms (*Eisenia* *fetida*) using LC–MS, ^1^H NMR based targeted metabolomics^28^. Several studies have highlighted the potential of metabolomics as an effective toxicological indicator. These investigations illustrate the power of metabolic profiling and metabolomics to understand and ascertain the biochemical alterations in organisms emerging from diverse environmental stresses^29,30^. Therefore, through comprehensive metabolic profiling, we can better understand and interpret the extent to which the earthworms are affected by different environmental invectives.

While previous studies have mainly focused on a single compound (pesticide) exposure, knowledge of exposure to combination of pesticides with different concentrations is currently lacking. Hence, this study represents an important transition from single pesticide exposure to a mixture of pesticide exposure on earthworms. With this background, the present work was designed to profile the metabolic changes in earthworms treated with three different pesticides chlorpyrifos (CHL), cypermethrin (CYP), Glyphosate (GLY), and Combined-(C), for putative identification of stress-specific metabolites, gas chromatography-mass spectrometry (GC–MS) based untargeted metabolomics was used.

## Materials and methods

### Chemicals and reagents

In this investigation following chemicals were used and all were obtained from Sigma Aldrich. Chlorpyrifos (45395), cypermethrin (36128), glyphosate (89432) pesticides, *N*-Methyl-*N*-trimethylsilyl trifluoroacetamide (MSTFA) (69479), Methoxyamine hydrochloride, methanol, and hexane.

### Soil preparation

In the present research, all the soils were prepared following the standard guidelines recommended by the Organization for Economic Co-operation and Development (OECD)^31^. The attributes of the artificial soils (AS) were as: quartz sand (70%), Kalonite (20%), peat moss (10%), pH (6.0 ± 0.5), and moisture content 35%.

### Control & treated group

**Figure 1:**
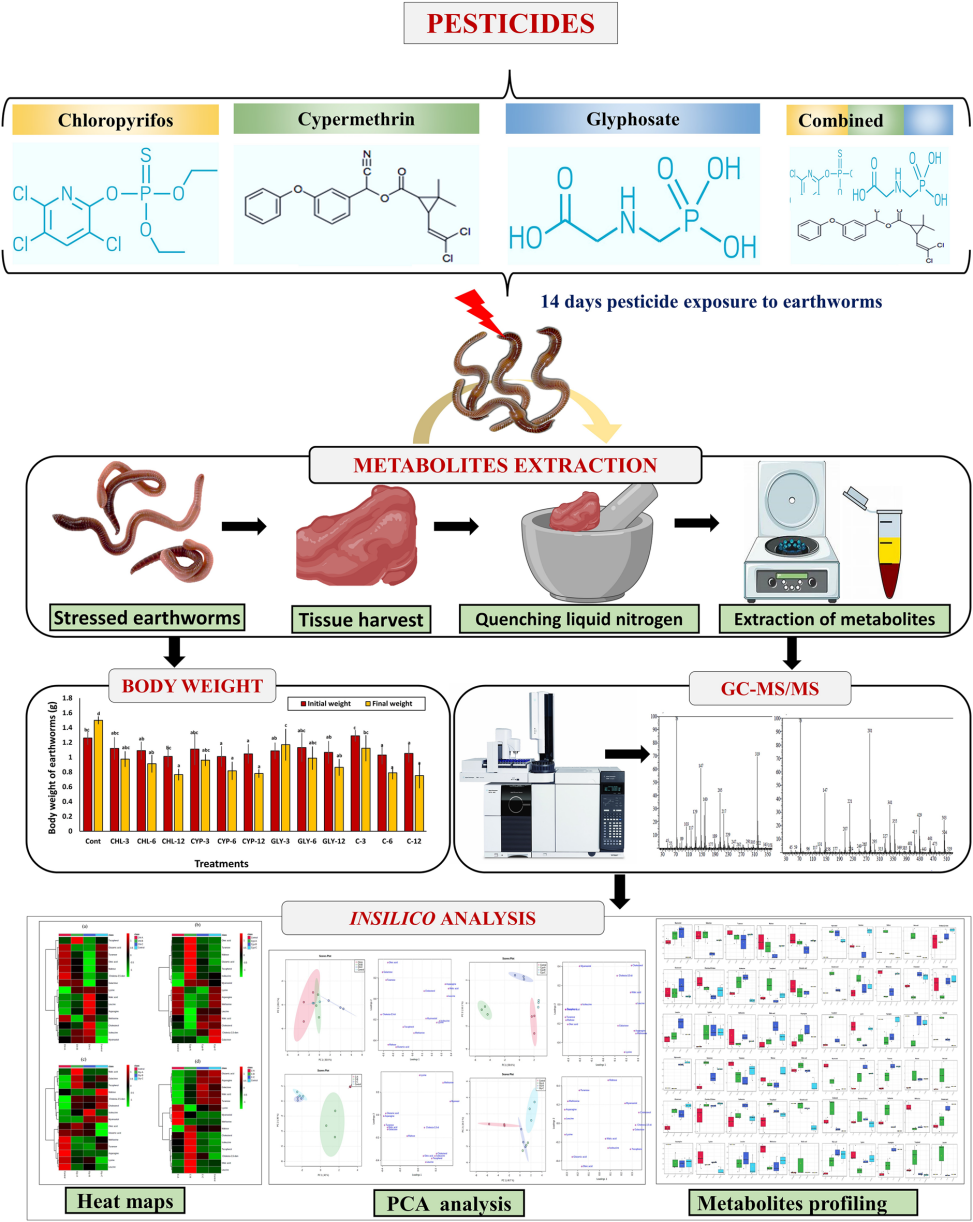
Workflow of the whole experiment.

The experiment was carried out as previously described^24,32,33^. Only well-developed clitellated adult worms sensitive to external stimuli were chosen. The test worms (*Eudrilus* *eugeniae*, accession number KX832073) were obtained from the worm-culture bed established at University Campus, DHSGU Sagar, Madhya Pradesh, India, and placed in artificial soil (AS) at 20 ± 3 °C to acclimatize for 2 weeks in a BOD incubator. One (1) kilogram of soil substrate was placed into 2 kg earthern pots and thoroughy mixed with sub-lethal concentrations (0, 3, 6, and 12 mg kg^−1^) of chlorpyrifos-CHL, cypermethrin-CYP, and glyphosate-GLY alone and in combination^24,34,35^. Five mature worms with a well-developed clitellum were weighted and added as a batch to each pot (N = 5 in each group), and the experiment was conducted for 14 days as per OECD guidelines^32^. On the 14 days of the experiment, earthworms were removed, washed, and weighed. The earthworms were then depurated on wet filter paper for two days to purge the gut contents, and the gut-cleansed earthworms were again rinsed and snap-frozen in liquid nitrogen, respectively lyophilized, and kept at -80 °C until use. The workflow of the whole experiment is given in Fig. 1.

### Earthworm metabolite extraction and derivatization

The metabolite extraction and derivatization were performed by following^36^, with slight modification. The lyophilized worms were immediately cut into pieces and homogenized. To the homogenized tissues, 1 ml of ice-cold solvent (methanol; 80%) was added, and the reaction mixture was vortexed for approximately 2 min, followed by sonication and centrifugation at 10,000 rpm at 4 °C for 10 min. Next, the supernatant was transferred to a new 2 mL Eppendorf tube for lyophilization to remove the organic phase. The dried samples were redissolved in 90 μL of 20 mg/mL *O*-methoxyamine hydrochloride in pyridine vortexed vigorously and incubated in a heating block at 70 °C for 40 min. Then, 150 μL of MSTFA was added, followed by derivatization at 70 °C for 1 h. Finally, the samples were diluted, and the volume was made up to 800 μL with HPLC-grade hexane for metabolic analysis using GC–MS.

### Gas chromatography–mass spectroscopy (GC–MS) instrumentation and data acquisition

Metabolite profiling of earthworm tissue extracts was performed on Nexis GC-2030 (Shimadzu). Agilent J&W DB-5MS capillary column (30 m × 0.250 mm i.d., 0.25 μm film thickness) consisting of a cross-linked stationary phase of 5% phenyl and 95% methyl polysiloxane, with injection temperature being set at 250 °C was used to separate the metabolites. Helium used as carrier gas was maintained at a flow rate of 1.1 mL min^−1^. The ion source, injector, and interface temperatures were set at 220, 250, and 290 °C, respectively. The initial column temperature was kept at 65 °C for 2 min, then increased to 230 °C at the rate of 6 °C min^−1^ and finally to 290 °C at the rate of 10 °C min^−1^, where it was held for 20 min. The detection was achieved using mass detection (MS) in electronic impact (EI) mode at 70 eV. The derivatized sample volume of 1 μL was injected for analysis, and the injection was carried out via splitless mode and scanned under the full mode of *m/z* 45–800 range.

### Statistical analysis

The resulting total ion chromatograms (TICs) obtained from the GC–MS runs were examined using web-based XC-MS software (https://xcmsonline.scripps.edu/)^37^. The individual peaks of the identified metabolites were compared with their corresponding spectrum via the NIST library, a public database. The preprocessed data sets were arranged in a three-dimensional matrix combining a set of metabolites and samples and auto-scaled for normalization. For multivariate statistical analysis, the resulting scaled data sets were uploaded to Metaboanalyst (v 4.0)^38^. Results were obtained, including one-way ANOVA to determine significantly altered metabolites, and PCA was performed to explore the clustering behaviour of essential metabolites. Pearson's coefficient correlation test was performed to evaluate the relationships between different metabolites and chronic exposure to pesticides using SPSS 21 (SPSS Inc./IBM Corp., Chicago, IL, USA). In addition, ANCOVA analysis was performed by Stata V.15 (StataCrp.2017)^39^.

## Results and discussion

### Body weight changes

During 14 days of exposure time (CHL, CYP, GLY, and C; 3, 6, and 12 mg kg^−1^), the survival rates of the worms in the control and the treated groups were recorded at 100%. No mortality occurred in any treatments, and none of the earthworms was found on the surface, reflecting that the tests were conducted robustly following the OECD standards^32^. Earthworms showed a significant reduction in body weight after exposure to pesticides (*p* < 0.05) (Table 1). In the control group, the average body weight of the worms increased from 1.26 to 1.5 g during 14 days (Table 1). However, a significant decrease in the body weight of worms was observed in all the treatment groups (*p* < 0.05) (Table 1). Earthworms in artificial soil (AS) treated with glyphosate at different concentrations lost less weight (~ 22), followed by chlorpyrifos (~ 24%), cypermethrin (~ 25) and combined (~ 34%) treatments (Table 1). Results also showed an increase in weight loss with increased concentration of pesticides from 3 to 12 mg kg^−1^ (Table 1). Moreover, ANCOVA analysis also showed that pesticide exposure significantly influenced the weight of worms and that the highest weight loss was found in the combined group, followed by cypermethrin, chlorpyrifos and glyphosate (Table S1 and Fig. S1). The physical symptoms that appeared on the earthworms include body coiling, fragmentation, abnormal swelling, and mucous secretion.

**Table 1:** Effect of selected pesticides (CHL, CYP, GLY, and C) on body weight (g) of earthworms.

Weight (g)	Initial weight (g)	Final weight (g)
Control	1.26 ± 0.09bc	1.5 ± 0.049d
CHL-3	1.12 ± 0.14abc	0.97 ± 0.10abc
CHL-6	1.09 ± 1.17ab	0.89 ± 0.11ab
CHL-12	1.01 ± 0.14a	0.76 ± 0.07a
CYP-3	1.11 ± 0.21abc	0.96 ± 0.07abc
CYP-6	1.01 ± 0.13a	0.82 ± 0.11a
CYP-12	1.04 ± 0.12a	0.74 ± 0.05a
GLY-3	1.17 ± 0.10ab	1.10 ± 0.21c
GLY-6	1.13 ± 0.19abc	0.98 ± 0.15abc
GLY-12	1.11 ± 0.14ab	0.86 ± 0.11ab
C-3	1.09 ± 0.07c	0.87 ± 0.17bc
C-6	1.03 ± 0.12a	0.78 ± 0.08a
C-12	1.05 ± 0.11a	0.69 ± 0.16a

Values represent the mean of five replicates ± SD (n = 5). Letters represent significant differences between treatments within a column according to Duncan’s multiple range test (*p* < 0.05).

### GC–MS based metabolic profiling of earthworms

**Figure 2:**
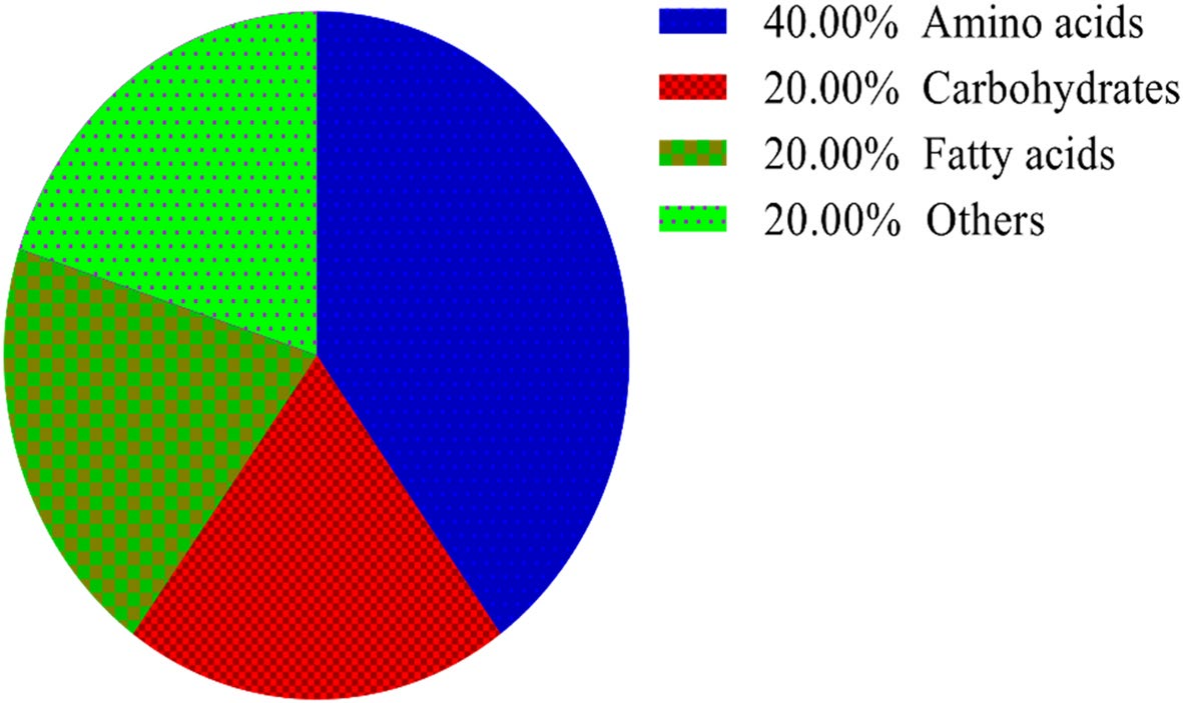
Distribution of the metabolites identified in earthworms exposed to different concentrations (3, 6 and 12 mg kg^−1^) of chlorpyrifos, cypermethrin, glyphosate individually and in combination. The pie chart shows the percentages of the identified metabolite classes.

**Figure 3:**
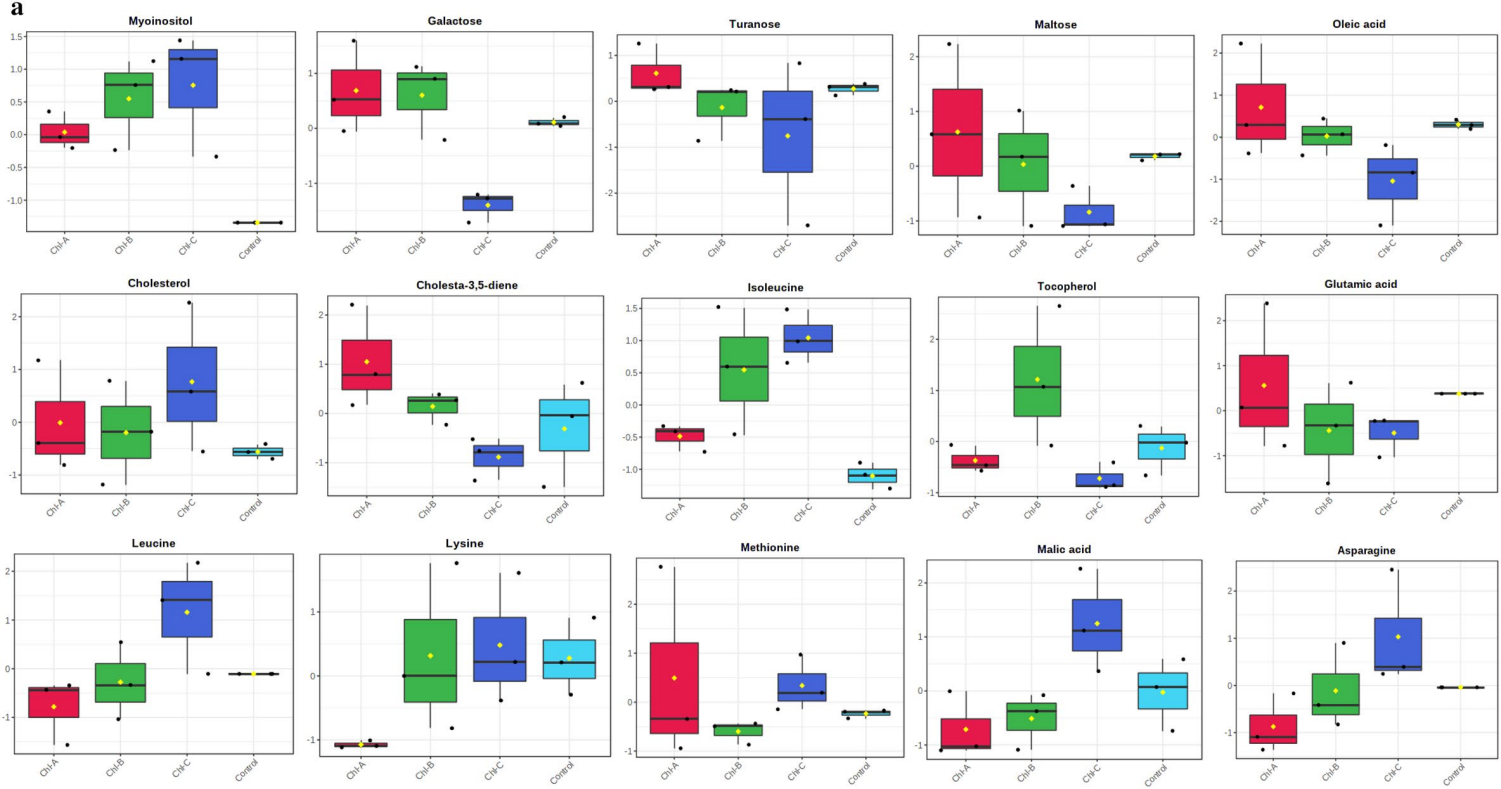

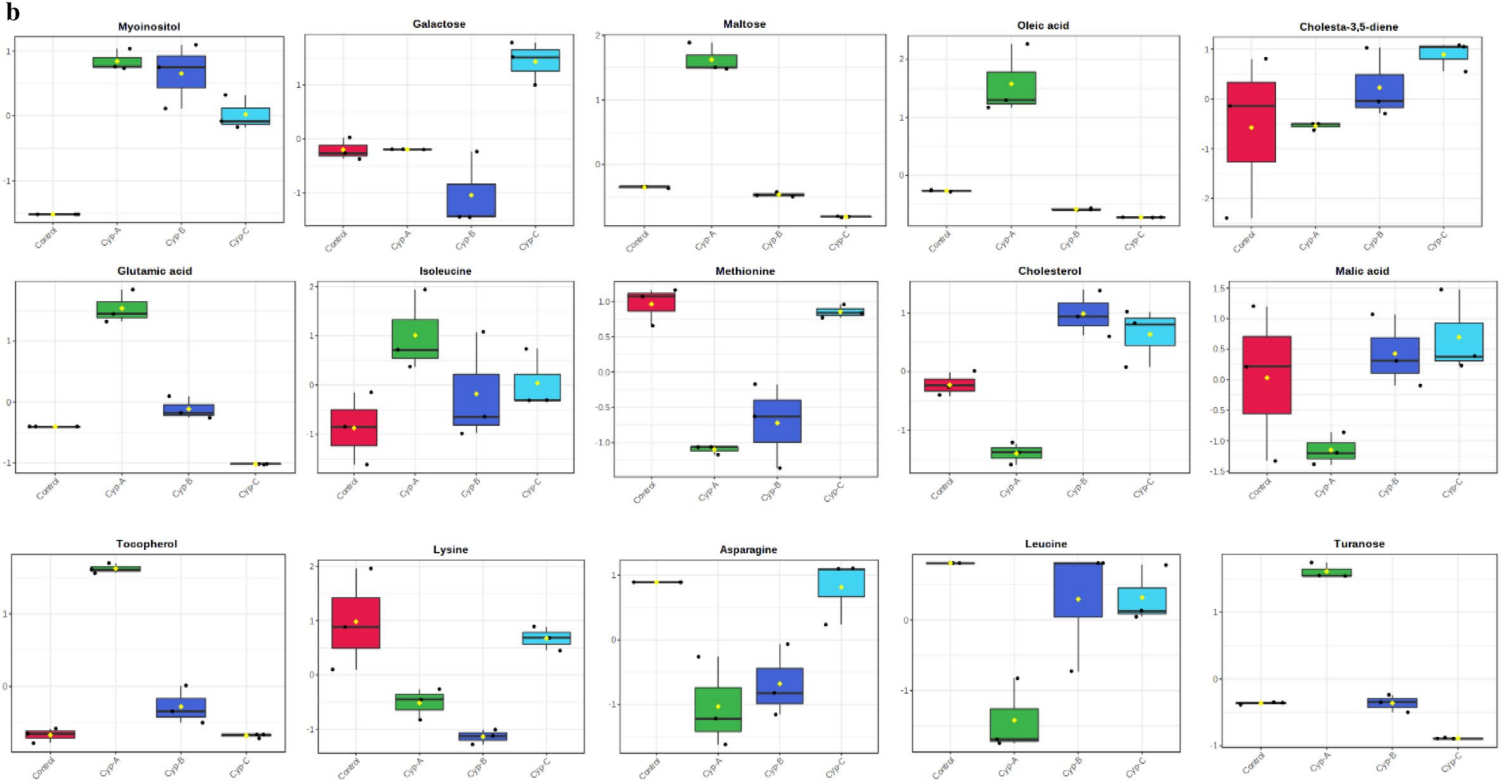

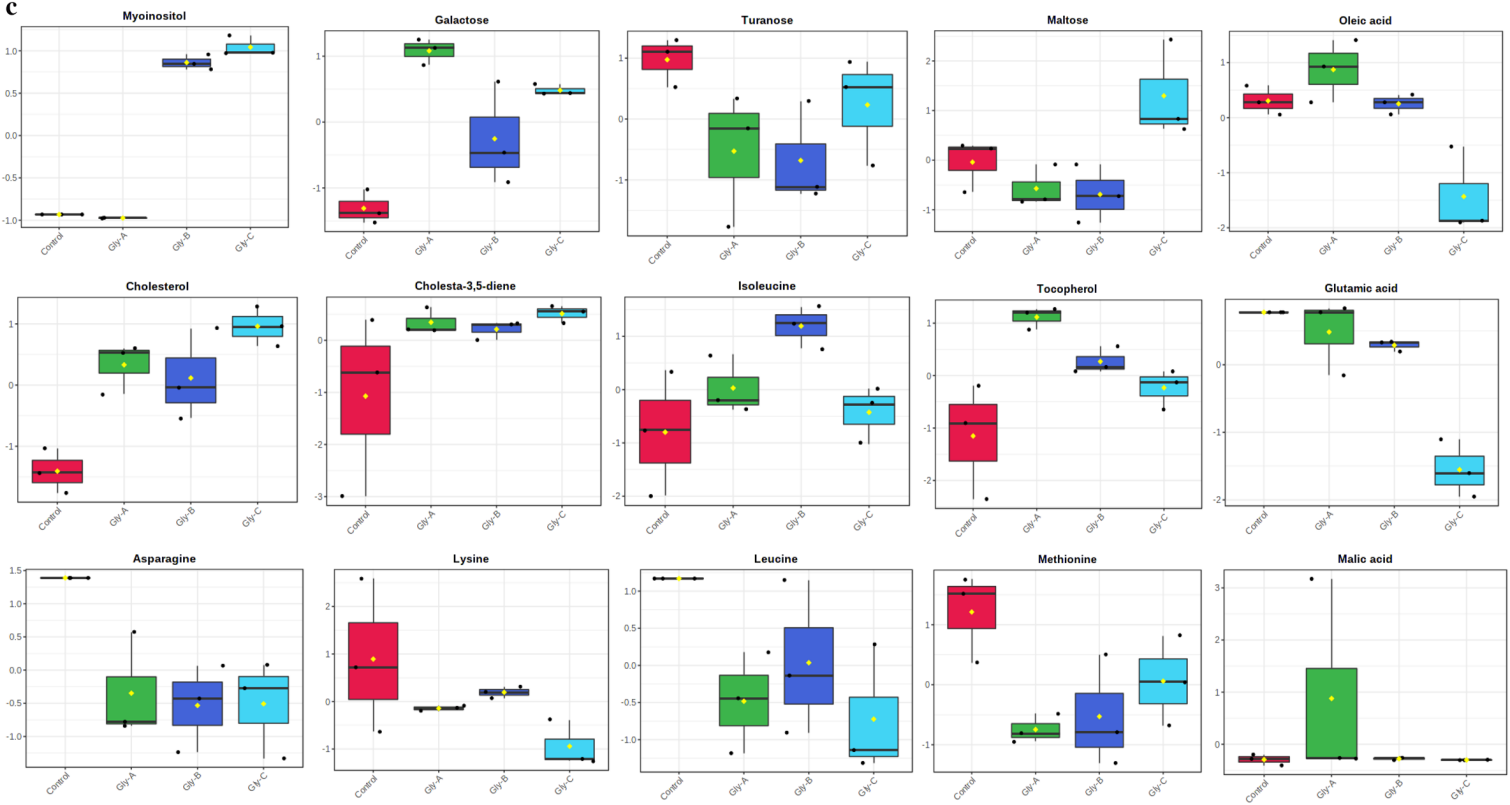

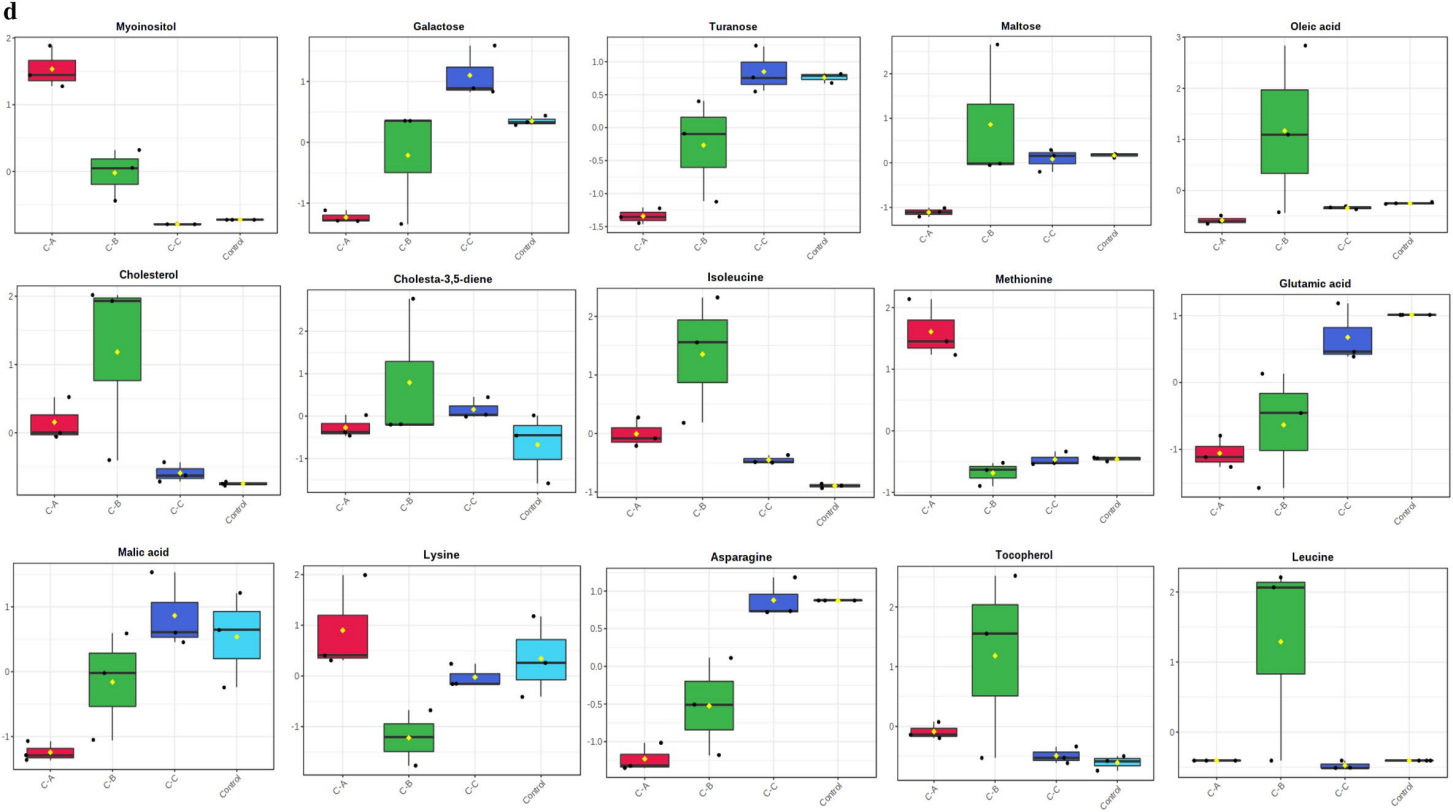
(**a**) Earthworm metabolite responses to chlorpyrifos exposure; control, Chl-A = 3 mg kg^−1^, Chl-B = 6 mg kg^−1^, Chl-C = 12 mg kg^−1^. (**b**) Earthworm metabolite response to cypermethrin exposure; control, Cyp-A = 3 mg kg^−1^, Cyp-B = 6 mg kg^−1^, Cyp-C = 12 mg kg^−1^. (**c**) Earthworm metabolite response to glyphosate exposure; control, Gly-A = 3 mg kg^−1^, Gly-B = 6 mg kg^−1^, Gly-C = 12 mg kg^−1^. (**d**) Earthworm metabolite response to combined exposure; control, C–A = 3 mg kg^−1^, C–B = 6 mg kg^–1^, C–C = 12 mg kg^−1^.

Out of the 28 metabolites, only those were selected that were found across all treated and control groups (Table 2), and all these metabolites were identified based on their maximum matching probability using the standard NIST Mass Spectral Library. The identified metabolites were categorized into amino acids (40%), sugars (20%), fatty acids (20%) and other acids and vitamins (20%) (Fig. 2 and Table 2). Following an investigation, the amino acids consisted mainly of leucine, glutamic acid, asparagine, isoleucine, lysine and methionine. The carbohydrates consisted mainly of galactose, turanose, and maltose, while the fatty acids included cholesterol, cholesta-3,5-diene and oleic acid. Among other acids and vitamins were myoinositol, malic acid and tocopherol (Table 2). The normalized concentrations of the identified metabolites in the pesticide were dose-dependent i.e., chlorpyrifos (Fig. 3a), cypermethrin (Fig. 3b), glyphosate (Fig. 3c), and combined (Fig. 3d).

**Table 2:** Represents the difference class of metabolites found to be significant between the control and treated groups (chlorpyrifos-CHL, cypermethrin-CYP, glyphosate-GLY and combined-C).

	Metabolites	CHL (%)	CYP (%)	GLY (%)	Combined (%)
Carbohydrates	Galactose	~ 91.48	~ 85.66 (*p* < 0.05)	~ 19.44 (*p* < 0.05)	~ 93.20
	Turanose	~ 79.14	~ 90.28 (p < 0.05)	~ 46.22	~ 95.04 (*p* < 0.05)
	Maltose	~ 81.14	~ 89.70 (*p* < 0.05)	~ 40.63% *(p* < 0.05)	~ 92.36
Amino acids	Leucine	~ 68.53	~ 90.20 (*p* < 0.05)	~ 47.37	~ 75.53
	Glutamic acid	~ 76.28	~ 91.81% (*p* < 0.05)	~ 41.88 (*p* < 0.05)	~ 91.8 (*p* < 0.05)
	Asparagine	~ 67.60	~ 89.15 (*p* < 0.05)	~ 45.20 (*p* < 0.05)	~ 94.20 (*p* < 0.05)
	Isoleucine	~ 26.28	~ 78.09	~ 29.51	~ 66.52 (*p* < 0.05)
	Lysine	~ 84.28	~ 92.20 (*p* < 0.05)	~ 49.22	~ 91.90 (*p* < 0.05)
	Methionine	~ 75.53	~ 92.27 (*p* < 0.05)	~ 48.69	~ 89.81 (*p* < 0.05)
Fatty acids	Cholesterol	~ 63.44	~ 91.56 (*p* < 0.05)	~ 20.84 (*p* < 0.05)	~ 22.51 (*p* < 0.05)
	Cholesta-3,5-diene	~ 82.99	~ 86.11	~ 17.06	~ 69.2 (*p* < 0.05)
	Oleic acid	~ 82.01	~ 93.47 (*p* < 0.05)	~ 38.75 (*p* < 0.05)	~ 87.96 (*p* < 0.05)
Others	Myoinositol	~ 83	~ 41.64 (*p* < 0.05)	~ 68.25 (*p* < 0.05)	~ 81.71 (*p* < 0.05)
	Malic acid	~ 73.57	~ 89.34	~ 63.00	~ 93.37 (*p* < 0.05)
	Tocopherol	~ 85.09	~ 47.26 (*p* < 0.05)	~ 9.46 (*p* < 0.05)	~ 36.98 (*p* < 0.05)

**Figure 4:**
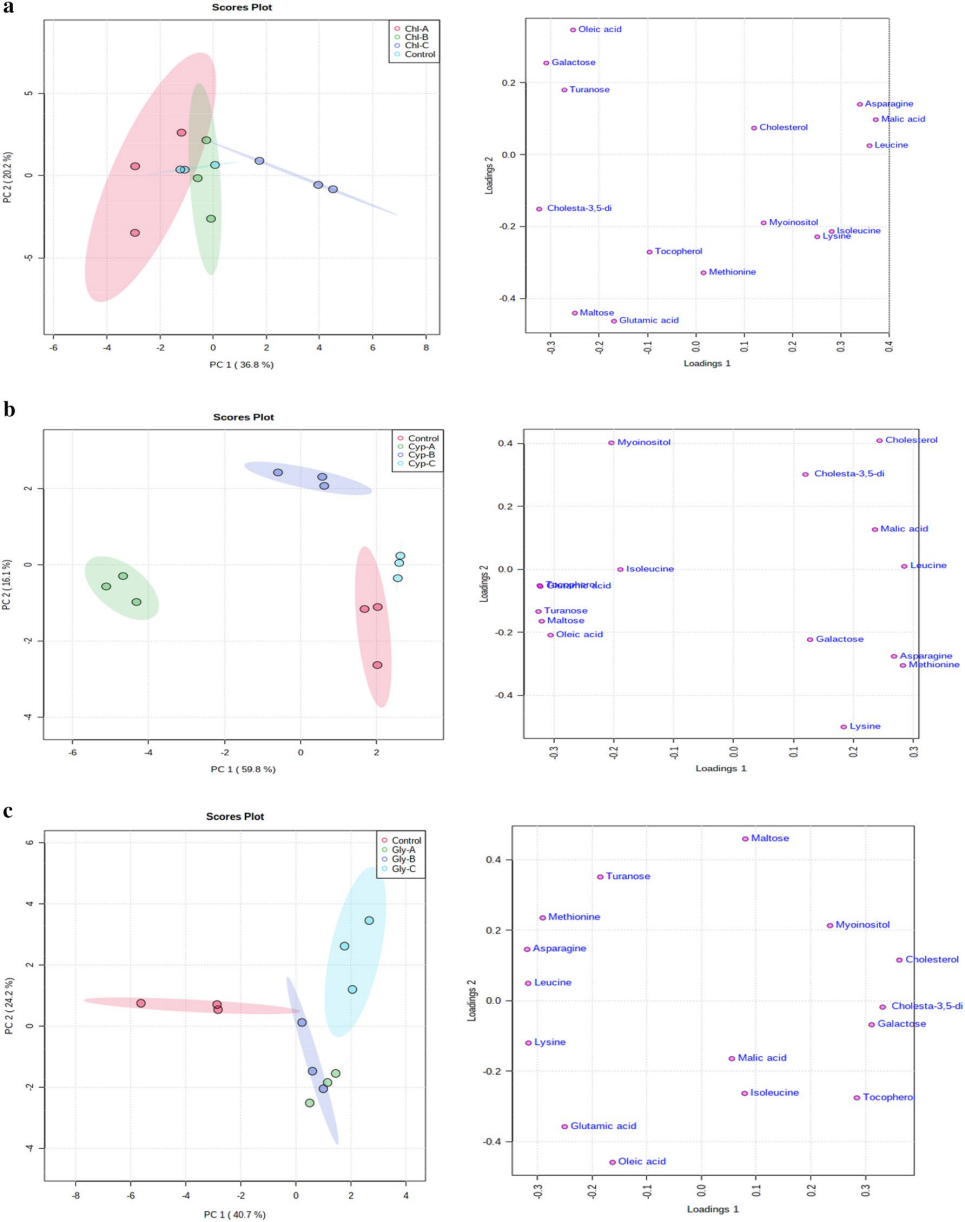

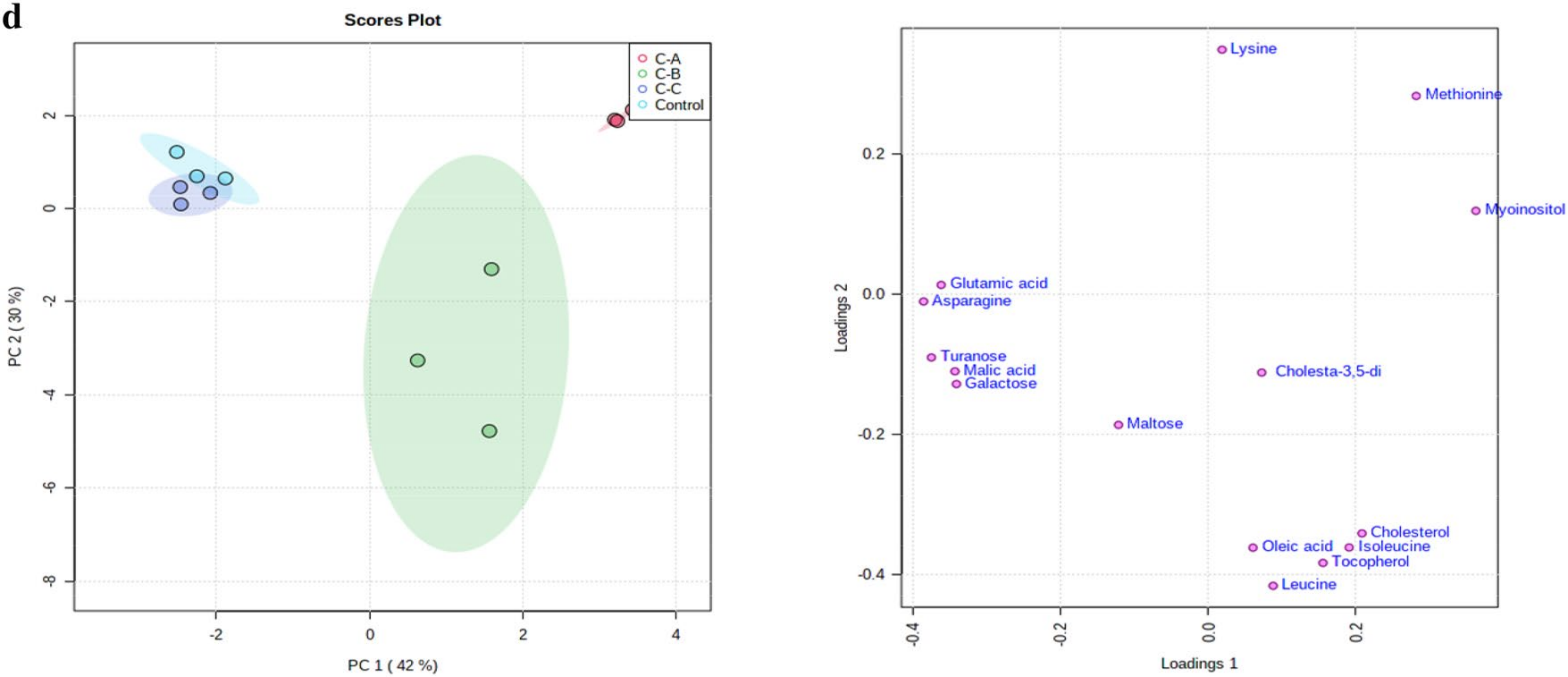
(**a**) PCA score and loading plots of control and chlorpyrifos treated worms (Chl-A = 3 mg kg^−1^, Chl-B = 6 mg kg^−1^, Chl-C = 12 mg kg^−1^). (**b**) PCA score and loading plots of control and cypermethrin treated worms (Cyp-A = 3 mg kg^−1^, Cyp-B = 6 mg kg^−1^, Cyp-C = 12 mg kg^−1^. (**c**) PCA score and loading plots control and glyphosate treated worms (Gly-A = 3 mg kg^−1^, Gly-B = 6 mg kg^−1^ and Gly-C = 12 mg kg^−1^). (**d**) PCA score and loading plots control and combined dose treated worms (C–A = 3 mg kg^−1^, C–B = 6 mg kg^−1^ and C–C = 12 mg kg^−1^).

To reduce the data to low dimensional space, multivariate analysis in terms of PCA analysis was carried out on the obtained data sets of control, chlorpyrifos, cypermethrin, glyphosate, and combined groups. The results of the PCA plots were displayed as score plots, indicating the scattering of samples, thus suggesting that similar metabolomic compositions when clustered together and compositionally diverse metabolomic compositions when separated. The unsupervised PCA model revealed the general structure of the data, in which two principal components showed a cumulative variance of 57%, with PC1 explaining 36.8% and PC2 20.2%, respectively, in the total variance for chlorpyrifos (Fig. 4a). While in the case of cypermethrin, the total variance was 75.9%, with the foremost PCAs explaining 59.8% PC1 and 16.1% PC2 (Fig. 4b). Similarly, in the case of glyphosate and combined groups, the total cumulative variance of the obtained data sets was 64.9% and 72%, with PC1 explaining 40.7% and 42% and PC2 explaining 24.2% and 30%, respectively, in the total variance (Fig. 4c, d), as shown in the figure, four different clusters are identified in the PCA score plot. Furthermore, from the score plots, a clear data separation was evident among the control and treatment groups and the separation was more significant with increasing concentration. The results showed that the metabolic responses to pesticide exposure were concentration-dependent (Fig. 4a–d).

Similarly, loading plot analysis was performed to understand the metabolic patterns. The loading plots of the first two principal components and the identified metabolites are shown in (Fig. 4a–d). In the case of chlorpyrifos, most metabolites, including asparagine, malic acid, leucine, isoleucine, lysine, myoinositol, cholesterol, methionine and tocopherol were associated with PC1 and hence had a positive correlation with PC1. In contrast, glutamic acid, maltose, cholesta-3,5-diene, turanose, oleic acid and galactose positively correlate with PC2 (Fig. 4a). While in the case of cypermethrin, the metabolites associated with PC1 include cholesterol, cholesta-3,5-diene, malic acid, leucine, galactose, lysine, asparagine, methionine and those associated with PC2 include isoleucine, maltose, turanose, myoinositol, tocopherol, glutamic acid, and oleic acid (Fig. 4b).

Similarly, glyphosate metabolites such as maltose, myoinositol, cholesterol, cholesta-3,5-diene, galactose, tocopherol, malic acid and isoleucine had a positive correlation with PC1 and a negative correlation with PC2, on the other hand, asparagine, methionine, leucine, lysine, turanose, glutamic acid and oleic acid were positively associated with PC2 (Fig. 4c). Again, in case of combined treatments the metabolites lysine, methionine, myoinositol, cholesta-3,5-diene, cholesterol, isoleucine, tocopherol, leucine, oleic acid showed a positive correlation with PC 1 and a negative correlation with PC2, while on the other hand metabolites such as maltose, galactose, malic acid, turanose, asparagine and glutamic acid showed a positive with PC2 and a negative correlation with PC1 (Fig. 4d). Overall, in the present study, score and loading plot analysis revealed a cumulative variation between different treatment groups, indicating apparent metabolic alterations in worms exposed to different pesticides.

### Heatmap analysis

**Figure 5:**
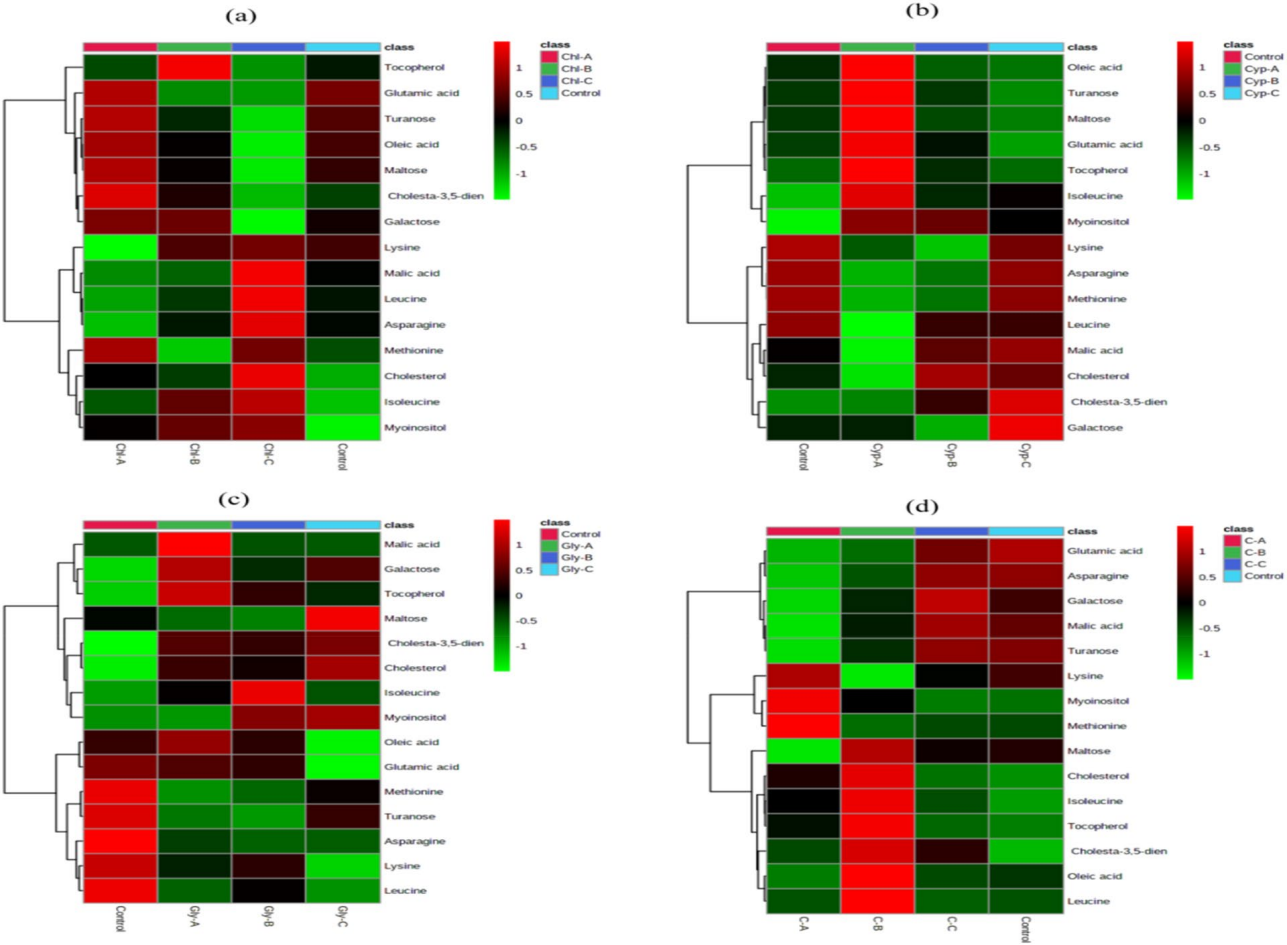
Heat map of the differentially identified metabolites in earthworm tissue extracts treated to (**a**) chlorpyrifos (Chl-A = 3 mg kg^−1^, Chl-B = 6 mg kg^−1^ and Chl-C = 12 mg kg^−1^) (**b**) cypermethrin (Cyp-A = 3 mg kg^−1^, Cyp-B = 6 mg kg^−1^ and Cyp-C = 12 mg kg^−1^) (**c**) glyphosate (Gly-A = 3 mg kg^−1^, Gly-B = 6 mg kg^−1^ and Gly-C = 12 mg kg^−1^) and (**d**) combined (C–A = 3 mg kg^−1^, C–B = 6 mg kg^−1^ and C–C = 12 mg kg^−1^.

Next, heatmap analysis was adopted to demonstrate the gradient changes of the identified metabolites between the control and the pesticide (CHL, CYP, GLY and C) treated groups and the analysis was performed based on the normalized data set of metabolites in earthworm tissue extracts. Heat map allows the visualization of large multidimensional data sets and the identification of metabolic patterns and trends under different experimental conditions. Figure 5a–d, shows the heat maps constructed from earthworm tissue of control and pesticide-exposed worms (Chl, Cyp, Gly and Combined-C).

Data is presented in a grid with columns representing different pesticides and their concentrations and rows representing metabolites. The color intensity of each cells means metabolic regulation in response to pesticide dose. The use of HCA (hierarchical clustering analysis) differentiated the metabolites into clusters of different regulatory trends, with metabolites in red showing a significant increase while those in green indicated a substantial decrease in concentration. From the heatmap analysis, it is clear that the concentration of metabolites such as oleic acid, lysine, glutamic acid, leucine, asparagine, methionine, malic acid, turanose maltose, cholesta-3, 5-diene, galactose, cholesterol, and Tocopherol in earthworm extracts decreased in the treated groups. In contrast, the concentration of Myo-inositol and isoleucine increased in a pesticide-treated group compared to the control groups (Fig. 5a–d).

### The changing trend for key metabolites across the different groups

**Figure 6:**
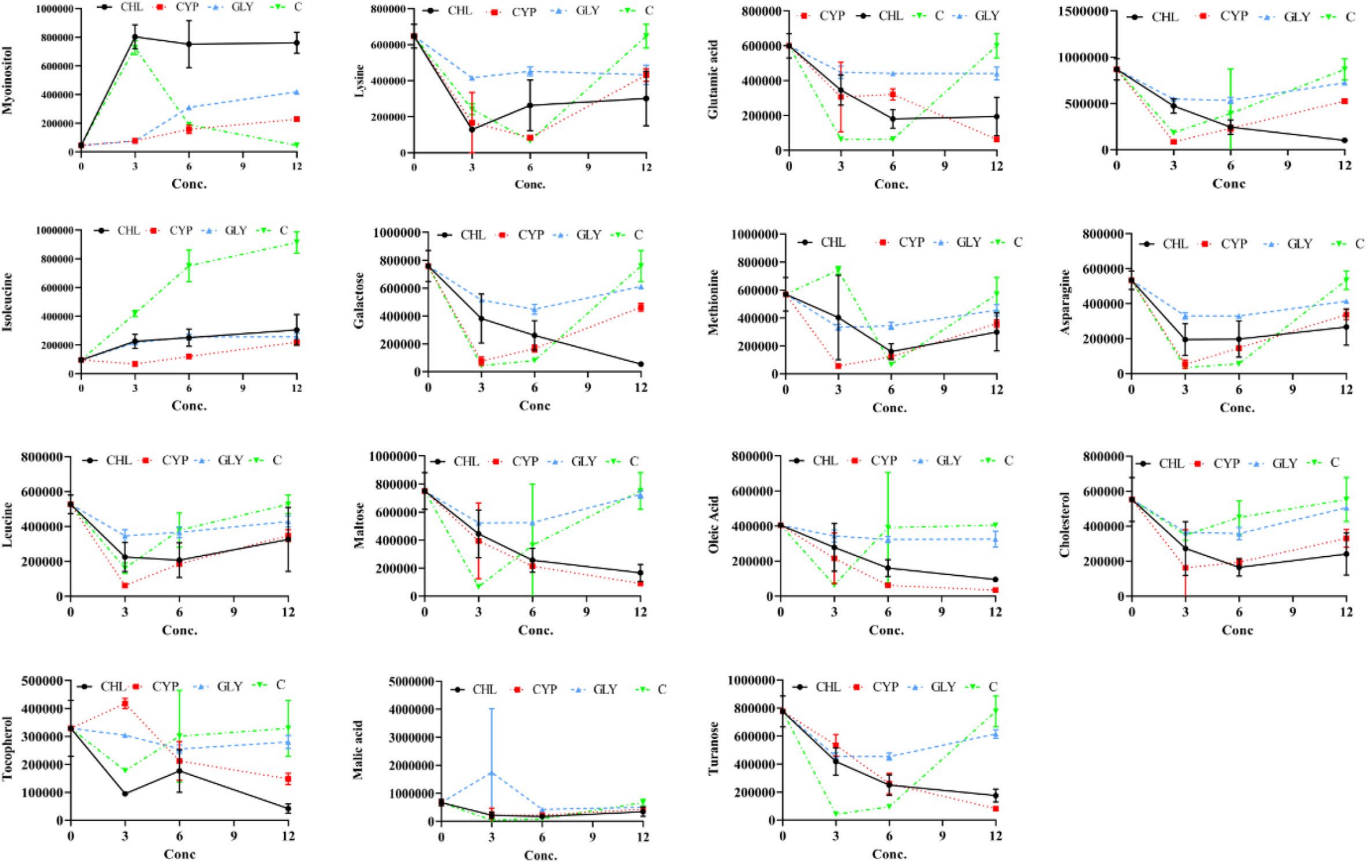
Plots showing the concentration of different metabolites against the different concentrations (3, 6 and 12 mg kg^−1^) of chlorpyrifos, cypermethrin, glyphosate and combined. The X-axis represents the dose concentration of pesticides and Y-axis the peak area of identified metabolites.

According to the peak area of each metabolite (where each peak stands for the relative concentration of a metabolite), we plotted the metabolites against different pesticide concentrations (Fig. 6). Among the identified metabolites, for those who decreased upon exposure to combined dose (C) of pesticides were oleic acid (~ 87.96%; *p* > 0.05), lysine (~ 91.90%; *p* < 0.05), glutamic acid (~ 91.8%; *p* < 0.05), leucine (~ 75.53%; *p* > 0.05), asparagine (~ 94.20%; *p* < 0.05), methionine (~ 89.81%; *p* < 0.05), malic acid (~ 93.37%; *p* < 0.05), turanose (~ 95.04%; *p* < 0.05) maltose (~ 92.36%; *p* > 0.05) cholesta-3,5-diene (~ 69.2%; *p* < 0.05), galactose (~ 93.20%; *p* > 0.05) cholesterol (~ 22.51%; *p* < 0.05), tocopherol (~ 36.98%; *p* < 0.05), and metabolites myoinositol (~ 81.71%; *p* < 0.05) and isoleucine (~ 66.52%, *p* < 0.05) were increased.

This study observed a significant positive correlation between the treated and the control groups except CHL-3, CHL-6; CHL12, where the data showed a positive correlation, but the difference were not statistically significant (Table 3). However, between the treated group CHL-3 was significantly associated with CHL-6 (*p* < 0.001), CHL-12 (*p* < 0.05); CYP-6 (*p* < 0.05), GLY-6 (p < 0.01); GLY-12 (*p* < 0.01) which may suggest a positive association or synergistic effect in earthworm when treated in combination. Similarly, CHL-6 group showed a significant positive association with CHL-12 (*p* < 0.001); C-3 (*p* < 0.001); C-12 (*p* < 0.05. Likewise, CHL-12 group showed a significant positive association with C3 (*p* < 0.001); C-12 (*p* < 0.05). In the case of CYP-3 treated group, a significant positive association with observed CYP-6 & CYP-12 (*p* < 0.001), GLY-3 & C-3 (*p* < 0.05), again the CYP-6 group showed a significant positive correlation GLY-3 (*p* < 0.05), GLY-6 & GLY-12 (*p* < 0.001) (Table 3). Whereas in the case CYP-12 group, a significantly positive correlation was observed with GLY-6 & GLY-12 (*p* < 0.001), C-12 (*p* < 0.05). In the case of glyphosate treatment, GLY-3 group was positively associated with C-12 (*p* < 0.05) and GLY-6 with GLY-12 & C-12 (*p* < 0.001), whereas GLY-12 showed a significant positive correlation with C-12 (*p* < 0.001) (Table 3). Based on the results, we suggest that the significant positive correlation may be because of the synergistic effect in earthworms when treated in combination.

**Table 3:** Illustrates the correlation coefficients (Pearson’s) among chronic pesticide-treated earthworms to different concentrations (3, 6 and 12 mg kg^−1^) of chlorpyrifos, cypermethrin, and glyphosate individually and in combination.

	CONT	CHL-3	CHL-6	CHL-12	CYP-3	CYP-6	CYP-12	GLY-3	GLY-6	GLY-12	C-3	C-6	C-12
CONT	−												
CHL-3	0.829	–											
CHL-6	0.059	0.000***	–										
CHL-12	0.096	0.024*	0.000***	–									
CYP-3	0.036*	0.155	0.610	0.147	–								
CYP-6	0.001***	0.021*	0.684	0.641	0.001***	–							
CYP-12	0.024*	0.323	0.571	0.194	0.002**	0.957	–						
GLY-3	0.049*	0.712	0.458	0.955	0.026*	0.048*	0.189	–					
GLY-6	0.000***	0.044**	0.713	0.728	0.092	0.000***	0.008**	0.102	–				
GLY-12	0.000***	0.006**	0.256	0.778	0.131	0.000***	0.007**	0.145	0.000***	–			
C-3	0.025*	0.080	0.000***	0.000***	0.044*	0.172	0.196	0.147	0.125	0.422	–		
C-6	0.379	0.992	0.993	0.657	0.190	0.550	0.786	0.503	0.765	0.795	0.343	–	
C-12	0.000***	0.230	0.014*	0.021*	0.282	0.080	0.023*	0.038*	0.000***	0.000***	0.086	0.178	–

*Correlation is significant at 0.05 level (2 tailed).

**Correlation is significant at 0.01 levels (2 tailed).

***Correlation is significant at 0.001 level (2 tailed).

## Discussion

During the 14 days of the exposure experiment, the mean weights of worms in pesticide-treated groups were underweight compared to the control group (Table 1). Weight loss is an important indicator of environmental and physiological stress and is linked to the exposure time and the level of toxicity, as suggested by ANCOVA analysis (Fig. S1), and reported in other studies^40,41^. Reduced body weight may indicate reduced feeding by the earthworms, as reported by^42,43^. These authors noted that when the worms are exposed to different pesticides, they dig less, which reflects less food intake and hence less bowel content. In addition, the observed morphological changes, such as body coiling, fragmentation, and mucous secretion, are related to weight loss^44,45^. Weight loss may result from feeding inhibition and alterations in muscular functions elicited by organophosphate and pyrethroid pesticides, leading to difficult locomotion for the intoxicated worms and their relative incapability to feed themselves^40,46^.

Metabolomics allows high-throughput identification and quantification of low molecular weight molecules of a biological system at a specific point in time. The present study examined the metabolic alteration in earthworms (*Eudrilus* *eugeniae*) after exposure to sub-lethal concentrations of three pesticides in a single and combined manner. The multivariate analysis of the metabolites clearly showed that perturbations occurred after exposure to the candidate pesticides and that these alterations are concentration-dependent. The pesticides in the soil ecosystem enter the earthworm body through the gut and dermal routes, reach throughout the body, and induce toxicity and oxidative stress^46,47^. The common enzymes responsible for xenobiotic metabolism in earthworms include GST (glutathione-*S*-transferases), carboxylesterases, and cytochrome P450^26,48^. The morphological symptoms include body coiling and fragmentation, mucous secretion, abnormal swelling and bleeding, CSR (cell stress response), a defense reaction of cells to environmental strain that commonly causes damage or structural deformations to nucleic acids (DNA), proteins, and other macromolecules^49^. The altered metabolites in individual and combined exposure are the amino acids and carbohydrates important for protein synthesis, stress response and energy production in earthworms^50^. The obtained metabolites were identified as amino acids, sugars, fatty acids, vitamins, and other organic acids using the NIST library.

Myo-inositol, an organic osmolyte, is vital in maintaining osmotic balance and functions as a secondary messenger in cells^51,52^. Its phosphate derivatives have several functions, including synthesizing membrane phospholipids, signal transduction, metabolic flux and transcription, mRNA export, and translation^53^. In this study, an increase in myo-inositol level was observed in pesticide-treated worms compared to control worms, indicating disturbances in the metabolism of phosphatidylinositol phosphate upon exposure to selected pesticides suggesting myo-inositol as a sensitive biomarker for assessing pesticide toxicity in earthworms, similar results were reported by^26,54^.

Glucose is the primary and essential energy source of the body, including the central nervous system (CNS)^55^, and it participates in energy metabolism^56^. Under stress conditions, the high energy requirements of the brain can lead to higher glucose utilisation. In this study, carbohydrate metabolites were downregulated in both individual and combined exposure compared to the control group, probably signifying an increase in energetics under stress conditions. Our findings align with studies suggesting pesticide toxicity induces changes in carbohydrate metabolism^25,57,58^. Patterns of altered glycolysis and glucogenesis have also been reported in mice following malathion exposure^59^, atrazine exposure in daphnia^60^, carbofuran and endosulfan exposure in earthworms^61,62^ respectively and in goldfish exposed to pesticide butachlor^63^. Given the alterations in metabolites related to energetics across different taxa and pesticides, these metabolomic alterations may represent an adverse effect of pesticide exposure as observed in earthworms (*Eudrilus* *eugeniae*) in this study. Our results suggest that glucose, galactose, and lactose are the sensitive biomarkers for earthworms treated with organophosphate and pyrethroid pesticides.

Long-chain fatty acids (LCFA) or free fatty acids form the critical constituents of CNS and are vital for proper functioning^64^. Their incorporation in the nerve cell membranes of the brain occurs through developmental processes and contributes to the functional maturation of CNS. Although glucose is the primary and the major energy source for the central nervous system, however under metabolic stress, the immune cells can utilize alternative energy sources such as fatty acids^65,66^. A decreasing trend in fatty acids (oleic acid, cholesterol, Cholesta-3,5-diene) was observed in treated worms compared to control worms, indicating a higher intake of fatty acids as an alternative energy source. The extra energy expenditure for CNS may be because of the stress caused by chlorpyrifos, cypermethrin, and glyphosate exposure.

Glutamate is a major excitatory neurotransmitter and has an essential role in TCA cycle, memory, and learning. Any damage to nervous systems could alter glutamate levels, thereby resulting in toxic effects on neurons. In this study, the levels of glutamate were downregulated in both individual and combined exposure compared to the control group. In this study, the different variations in the levels of glutamate suggest that the mechanism of action of a single pesticide in earthworms may be different to that of the combined exposure of pesticides^67^. Isoleucine, a branched-chain amino acid, is important for various physiological functions in various taxa^68,69^. Isoleucine plays a significant role in immune function, including maintaining immune cells and organs and stimulating the secretion of immune molecules^70,71^. In this study, the up-regulation of isoleucine was observed in the earthworms treated with (CHL, CYP, GLY, and C) pesticides compared to the control. Our results are consistent with the earlier reports on earthworms and Pisces (*Clarias* *batrachus*) treated to the sub-lethal concentrations of carbofuran and phenanthrene^61,72,73^. Methionine, another key amino acid, plays a crucial role in the antioxidant defense system^74^ and may act as a bioindicator of oxidative stress^60^. Additionally, methionine is an essential part of the cysteine methionine metabolism pathway. Dysregulation of methionine in earthworms following pesticide exposure has profound impacts on the survival rate and disease resistance. Furthermore, amino acids like lysine, leucine, and asparagine were down-regulated in pesticide (CHL, CYP, GLY, and C) treated worms compared to control worms. This downregulation of these amino acids may be linked to the production of enzymes involved in fatty acid oxidation^75^. The variations in the levels of these amino acids between different treatment groups may also be attributed to the weight loss of worms because of the stress of the treated pesticides such as chlorpyrifos, cypermethrin, glyphosate, and combined^25,76,77^. The difference in toxicity levels and hence the alterations in key amino acid metabolites indicates the differences in mode of action and toxicity levels of these pesticides^78^. Another possible reason for the downregulation of these amino acids may be linked to the production of enzymes for the metabolism of these pesticides due to the activation of the detoxification strategy^79^. Amino acids play a key role in cellular metabolism, including protein synthesis, and therefore can be used to estimate the rate of protein synthesis^80^. Thus, the depletion of the key amino acid metabolites in pesticide-treated groups advocates a significant reduction in protein synthesis in treated earthworms.

Malic acid is an essential metabolite that plays a vital role as an acidity regulator in food and an important metabolite in the TCA cycle. The TCA cycle has two key functions; it involves some of the key intermediate compounds for synthesizing fatty acids and amino acids and the production of ATP, which is the energy source for various synthetic processes^81^. The downregulation of malic acid metabolite in earthworms treated with different doses of pesticides is possibly due to the scarcity of the intermediate compounds and energy in earthworms treated with pesticides (chlorpyrifos, cypermethrin, glyphosate, and combined). Similar results were also reported by Zhou et al.^82^. Downregulation of malic acid suggests inhibition in TCA cycle, which is the center of carbohydrate, protein, and lipid metabolism and is the primary metabolic process for supplying energy^27,83^. The difference in alteration between different pesticide groups may be associated with their modes of action and toxicity levels^84^.

Overall, pesticide exposure induced changes to basic but essential metabolites in key biological pathways, as observed in the present study. Multivariate analysis showed a clear molecular group response to pesticide exposure at the metabolic level. Identifying the metabolic perturbations in response to environmental stressors can identify the potential biomarkers of these stressors^25,85^. The integrated data sets indicated that pesticide exposure could cause neurotoxic effects, amino acid and energy mechanisms disorders, and osmotic balance in earthworms. These metabolites can be potential biomarkers to assess the toxicity of pesticides in earthworms. However, targeted metabolomic profiling here would offer much greater sensitivity and an in-depth description and our efforts in identifying the key biomarkers following combined pesticide exposure in earthworms and other soil invertebrates.

## Conclusion

Metabolomics, particularly environmental metabolomics, has emerged as a new discipline with the potential to link earthworm toxicity and the bioavailability of soil toxicants. To our knowledge, the present study is the first to evaluate earthworms' metabolomic response following exposure to three different pesticides. After 14 days of exposure, the body weight of the earthworm significantly reduced in the treated groups (*p* < 0.05). Metabolic profiling revealed that the metabolite alterations were more sensitive to higher pesticide concentrations and that the alterations were more prominent in combined exposure. The results showed that pesticides impaired the key biological pathways related to oxidative damage, energy deficiency, and liver and nervous functional disorders. Glutamic acid, oleic acid, lysine, leucine, asparagine, methionine, malic acid, turanose maltose, cholesta-3, 5-diene, galactose, may serve as potential biomarkers for soils contamination by pesticides. Overall, the present study suggests that environmental metabolomics has great potential to determine the mechanism of action and better understand the toxicological impacts of environmental contaminants.

## Supplementary Information

Supplementary Information.

## Data Availability

The datasets used and/or analyzed during the current study are available from the corresponding author upon reasonable request.
