# Supplementary material for: GC–MS based untargeted metabolomics reveals the metabolic response of earthworm (Eudrilus eugeniae) after chronic combinatorial exposure to three different pesticides
Source: Sci Rep. 2023 May 26;13:8583. doi: 10.1038/s41598-023-35225-1 (PMC10220075; doi:10.1038/s41598-023-35225-1)
Supplement: Supplementary file 1 — Supplementary Information. [file 41598_2023_35225_MOESM1_ESM.docx]

| **Tests of Between-Subjects Effects** | | | | | |
| --- | --- | --- | --- | --- | --- |
| Dependent Variable: Weight | | | | | |
| Source | Type III Sum of Squares | df | Mean Square | F | Sig. |
| Corrected Model | .503^a^ | 4 | .126 | 14.467 | <.001 |
| Intercept | 13.300 | 1 | 13.300 | 1529.533 | <.001 |
| Dose | .336 | 1 | .336 | 38.632 | <.001 |
| Group | .167 | 3 | .056 | 6.413 | <.001 |
| Error | .478 | 55 | .009 |  |  |
| Total | 45.230 | 60 |  |  |  |
| Corrected Total | .981 | 59 |  |  |  |
| a. R Squared = .513 (Adjusted R Squared = .477) | | | | | |

**Table S1. Univariate Analysis of Variance of different groups to weight**

**Group**

**GLY**

**CYP**

**CHL**

**C**

1.000

.800

.600

.400

.200

.000

**Estimated Marginal Means of Weight**

Covariates appearing in the model are evaluated at the following values: Dose = 7.00

Error bars: 95% CI

**Estimated Marginal Means**

**Figure S1: Pot showing the estimated marginal means of weight**
